# Acceptability of restrictions in the COVID-19 pandemic: a population-based survey in Denmark and Sweden

**DOI:** 10.3389/fpubh.2023.988882

**Published:** 2023-08-03

**Authors:** Per Nilsen, Ida Seing, Mandeep Sekhon, Thomas Kallemose, Tine Tjørnhøj-Thomsen, Nina Thórný Stefánsdóttir, Karsten Vrangbæk, Ove Andersen, Jeanette Wassar Kirk

**Affiliations:** ^1^Department of Health, Medicine and Caring Sciences, Linköping University, Linköping, Sweden; ^2^Department of Behavioral Sciences and Learning, Linköping University, Linköping, Sweden; ^3^Population Health Research Institute, St George’s University of London, London, United Kingdom; ^4^Department of Clinical Research, Copenhagen University Hospital, Amager and Hvidovre, Hvidovre, Denmark; ^5^National Institute of Public Health, University of Southern Denmark, Copenhagen, Denmark; ^6^Department of Public Health, University of Copenhagen, Copenhagen, Denmark; ^7^Department of Clinical Medicine, University of Copenhagen, Copenhagen, Denmark

**Keywords:** acceptability, compliance, COVID-19, restrictions, survey

## Abstract

**Introduction:**

Denmark and Sweden initially adopted different responses to the COVID-19 pandemic although the two countries share many characteristics. Denmark responded swiftly with many mandatory restrictions. In contrast, Sweden relied on voluntary restrictions and a more “relaxed” response during the first wave of the pandemic. However, increased rates of COVID-19 cases led to a new approach that involved many more mandatory restrictions, thus making Sweden’s response similar to Denmark’s in the second wave of the pandemic.

**Aim:**

The aim was to investigate and compare the extent to which the populations in Denmark and Sweden considered the COVID-19 restrictions to be acceptable during the first two waves of the pandemic. The study also aimed to identify the characteristics of those who were least accepting of the restrictions in the two countries.

**Materials and methods:**

Cross-sectional surveys were conducted in Denmark and Sweden in 2021. The study population was sampled from nationally representative web panels in the two countries, consisting of 2,619 individuals from Denmark and 2,633 from Sweden. The questionnaire captured key socio-demographic characteristics. Acceptability was operationalized based on a theoretical framework consisting of seven constructs and one overarching construct.

**Results:**

The respondents’ age and gender patterns were similar in the two countries. The proportion of respondents in Denmark who agreed with the statements (“agree” alternative) that captured various acceptability constructs was generally higher for the first wave than the second wave of the pandemic. The opposite pattern was seen for Sweden. In Denmark, 66% in the first wave and 50% in the second wave were accepting of the restrictions. The corresponding figures for Sweden was 42% (first wave) and 47% (second wave). Low acceptance of the restrictions, defined as the 25% with the lowest total score on the seven acceptability statements, was associated with younger age, male gender and lower education levels.

**Conclusion:**

Respondents in Sweden were more accepting of the restrictions in the second wave, when the country used many mandatory restrictions. In contrast, respondents in Denmark were more accepting of the restrictions in the first wave than in the second wave, implying an increased weariness to comply with the restrictions over time. There were considerable socio-demographic differences between those who expressed low acceptance of the restrictions and the others in both countries, suggesting the importance of tailoring communication about the pandemic to different segments of the population.

## 1. Introduction

Despite sharing many cultural, historical, political and economic characteristics (1), Denmark and Sweden initially adopted different responses to the COVID-19 pandemic (2). Denmark responded swiftly with many mandatory restrictions (3). In contrast, Sweden’s response was characterized by voluntary restrictions with an emphasis on people’s willingness to assume responsibility to comply with the restrictions (4). Sweden had a much higher incidence, hospitalization and mortality rates due to COVID-19 in the first two waves of the pandemic than Denmark and the other Nordic countries (5), which led to national and international critique about the country’s “relaxed” or “light touch” response (6).

The difference between the two countries was most apparent in the early period of the coronavirus pandemic when the Danish Government required lockdowns of parts of society and Sweden remained open. The restrictions in Denmark were possible due to a revision of the so-called Infectious Diseases Act, in which the Minister of Health was granted a number of powers to ensure containment of dangerous diseases (such as COVID-19). Sweden’s approach changed in response to increased rates of COVID-19 cases when the second wave of coronavirus hit in the autumn/winter 2020–2021. This led to rapid development of a Pandemic Law and adoption of many more mandatory restrictions, thus making Sweden’s response more similar to Denmark’s (4).

For mandatory and voluntary COVID-19 restrictions to be effective, they must be viewed as acceptable by the recipients in order to achieve compliance (7). Acceptability has been defined as a multifaceted concept that reflects the extent to which people delivering or receiving an intervention (e.g., a social distancing measure) consider it to be appropriate, based on anticipated or experienced cognitive and emotional responses to the measure (8). Acceptability is a necessary but not sufficient condition for compliance with an initiative aimed at influencing behaviours. If a restriction is considered acceptable, recipients are more likely to comply with the measure, thus increasing the likelihood of its effectiveness (9).

Some research has been conducted to investigate public support for restrictions to reduce the spread of the virus during the COVID-19 pandemic. A study by Jörgensen et al. (10) surveyed people in eight western countries, including Denmark and Sweden, using a broad approach to ask whether the respondents believed “the government conducts the policy necessary to handle the Corona virus.” However, even if a restriction is considered necessary, it may not be viewed as acceptable so compliance could still be limited. An Irish study by Lunn et al. (11) investigated different communication approaches to promote compliance with social distancing measures. The study was an online experiment, testing the acceptability of three hypothetical or actual behaviours during the pandemic (travel by public transport; allow children to play outside with friends; travel to their parent’s house for a cup of tea and a chat).

There is a lack of research regarding the overall acceptability of COVID-19 restrictions in countries that have used divergent responses to manage the pandemic. We do not know the extent to which the pandemic restrictions to reduce the spread of the virus in Denmark and Sweden were considered acceptable by the general public in these countries. There is also a paucity of information on whether there were differences in the acceptability of the restrictions between the two countries and how acceptability was associated with age, gender, education and occupation of individuals. Such information might be important for improved tailoring of messages regarding restrictions. To explore such issues, we used an analytical design with the same survey instrument in Denmark and Sweden. The aim of this study was to investigate and compare the extent to which the populations in Denmark and Sweden considered the COVID-19 restrictions during the first two waves of the pandemic to be acceptable. The study also aimed to identify the characteristics of those who were least accepting of the restrictions in the two countries.

## 2. Materials and methods

### 2.1. Study design and population

Cross-sectional surveys were conducted in Denmark and Sweden in 2021. The study population was sampled from web panels in Denmark and Sweden, administered by Enkätfabriken, a company that specializes in survey research (12). The panels have been assembled to be nationally representative of socio-demographic characteristics. Participants in the web panel are randomly recruited by telephone and agree to participate in a number of questionnaires. Participation in the web panel is compensated by means of a points system; points can then be exchanged for money or be donated to charity. The web panel is screened routinely to exclude inactive participants, that is, individuals who have not participated in a questionnaire within the last 12 months.

The total study population consisted of 5,252 individuals: 2619 from Denmark and 2,633 from Sweden. They were sampled to be representative of the age, gender and region of residence of the Danish and Swedish populations aged ≥18 years.

The reporting accounted for the contents of the STROBE (The Strengthening the Reporting of Observational Studies in Epidemiology) checklist (13), which is submitted as a Supplementary material.

### 2.2. Data collection

The survey data were collected by means of an electronic questionnaire that was distributed via the web panel in April 2021. The participants received a personalized code to make it possible to return to the questionnaire if they were not able to complete all questions in one sitting. All responses were kept on Enkätfabriken’s internal servers and were not accessible by the researchers behind this study until all participants had completed the questionnaire.

### 2.3. Think-aloud study

A think-aloud study was conducted with the aim of detecting potential problems in participants’ interpretations of various instructions, questions and response items in the questionnaire. The reliability and validity of self-report measures depend on participants interpreting and responding as intended by the researchers. The think-aloud method requires verbalization of thoughts that would normally be silent (14).

To conduct the think-aloud study, we used an opportunistic sample by recruiting nine individuals from the authors’ social circles, aged 17–71 years, five men and four women, five Danish citizens and four Swedish citizens. The participants provided written or oral informed consent and were given a paper-and-pen version of the planned questionnaire, with the following written instruction: “We are seeking to find out how the questions in this questionnaire about the COVID-19 pandemic are interpreted. Please fill in the questionnaire and think-aloud when doing so. By ‘thinking aloud’ we mean your thoughts, from reading a question until you have decided on a response. Please comment as if you were alone in the room and speaking to yourself.” Four of the authors administered the think-aloud study, each interviewing two or three persons. The researcher at each session took notes pertaining to each question of the questionnaire.

The notes from the nine think-aloud sessions were assembled by the first author of this study and discussed among the authors in a Zoom meeting. This process led to several modifications in the formulation of various questionnaire instructions, questions and response items. The changes were discussed and agreed upon by all authors at a Zoom meeting.

### 2.4. Contents of the questionnaire

The questionnaire consisted of three questions on socio-demographic characteristics: gender, education and occupation. Information about the respondent’s age was collected by Enkätfabriken.

Acceptability was operationalized based on a theoretical framework developed by Sekhon et al. (8). According to this framework, acceptability consists of seven constructs and one general acceptability construct. The seven constructs are defined as: (1) Affective attitude concerns how an individual feels about taking part in an intervention; (2) Burden is the perceived amount of effort that is required to participate in the intervention; (3) Perceived effectiveness is the extent to which the intervention is perceived as likely to achieve its purpose; (4) Ethicality is the extent to which the intervention has good fit with an individual’s value system; (5) Intervention coherence is the extent to which the participant understands the intervention and how it works; (6) Opportunity cost is the extent to which benefits, profits or values must be given up to engage in an intervention; and (7) Self-efficacy is the participant’s confidence that he or she can perform the behaviour(s) required to participate in the intervention (14). We constructed one response item, in the form of a statement, for each of the eight acceptability framework constructs (Table 1).

**Table 1:** Operationalization of acceptability components.

Acceptability construct	Definition (8, 14)	Definition when adapted to the study context	Statement used in the survey questionnaire
General acceptability item	The participants’ overall judgement of the acceptability of the intervention	The individual’s overall judgement of the acceptability of the restrictions	“I believe the restrictions to reduce the coronavirus spread were acceptable”
Affective attitude	How an individual feels about the intervention	How an individual feels about the restrictions	“I recognized the value of the restrictions to reduce the coronavirus spread”
Ethicality	The extent to which the intervention has a good fit with an individual’s value system	The extent to which restrictions have good fit with an individual’s value system	“I think it was immoral not to comply with the restrictions to reduce the coronavirus spread”
Coherence	The extent to which the participant understands how the intervention works	The extent to which the individual understands the restrictions and how they work	“I understood the purpose of the restrictions to reduce the coronavirus spread”
Perceived effectiveness	The extent to which the participant perceives the intervention to be likely to achieve its intended purpose	The extent to which the restrictions are perceived as likely to achieve their purpose	“I believe the restrictions to reduce the coronavirus spread were effective”
Self-efficacy	The participants’ confidence that they can perform the behaviour(s) required to participate in the intervention	The individual’s confidence that they can perform the behaviour(s) required to comply with the restrictions	“I was able to comply with the restrictions to reduce the coronavirus spread”
Burden (reversed item)	The amount of effort required to participate in the intervention	The amount of effort required to comply with the restrictions	“I made efforts to comply with the restrictions to reduce the coronavirus spread”
Opportunity costs (reversed item)	The benefits, values or profits that must be given up to engage with the intervention	The benefits, values or profits that must be given up to comply with the restrictions	“It was a great burden for me to comply with the restrictions to reduce the coronavirus spread”

The questionnaire also included two questions concerning trust (trust in how politicians and public health authorities handled the pandemic and in the information conveyed by a number of actors) and a question about individuals’ altered behaviours in response to the pandemic. Furthermore, the questionnaire included an open question that asked the respondents to provide suggestions regarding what they believe could have been done differently and/or better to reduce the spread of coronavirus. The question on trust, behaviour changes and the open question were not analysed for this study, but are part of another study.

The questionnaire was developed in Swedish before being translated into Danish. Due to the close similarity between the Swedish and Danish languages, there was no need for an elaborate back translation process. Instead, any linguistic uncertainties were handled by discussions among the Danish and Swedish authors. The Danish translation was adjusted until it fit well with the Swedish version. The Danish and Swedish authors jointly approved the final versions.

### 2.5. Statistical analysis

No prior sample size calculation was performed for the study. Demographic variables are presented as means with standard deviation or medians with interquartile range for continuous variables and as frequencies with percentages for categorical variables.

The seven statements concerning acceptability were posed in relation to the first wave of the pandemic (spring-autumn 2020) and the second wave (autumn/winter 2020-spring 2021). Responses were on a five-point Likert scale (from “do not agree” to “agree”). Statements for all constructs are positively correlated with acceptability (e.g., the higher the self-efficacy or perceived effectiveness, the more likely the restriction is perceived as acceptable) except for two reversed constructs, burden and opportunity costs, which are negatively correlated with acceptability (e.g., the larger the effort or sacrifices made to comply with restrictions, the less likely it is that the restriction was perceived as acceptable) (8).

In line with the guidance provided by Sekhon et al. (15), the generic acceptability item was included in the questionnaire to determine overall evaluations of acceptability of the restrictions. For analysis, we calculated the mean score (1, lowest level of acceptability; 5, highest level of acceptability) for this item. For the remaining seven acceptability items, a total score of acceptability was calculated by numbering the responses for each item 1 to 5, with 5 indicating the highest level of acceptability and 1 the lowest. The sum of the seven items thus ranged from 5 to 35. This score was then dichotomized as low/not low acceptability by allocating responses within each country in the lowest quartile (i.e., lowest 25%) of the total score to low acceptability, labelled “low accepters,” and all others to not low acceptability. Correlation for the seven acceptability items used in the total score and the total score itself with the generic acceptability item were estimated by Pearson’s correlation coefficient with the 95% confidence interval (CI).

The effects of demographic variables (age, gender, education and occupation) on low acceptability within Sweden and Denmark were analysed by logistic regression models for each wave and change by mixed logistic regression models. Models were fitted for each demographic variable with no additional variables for the total population and within each country.

Estimates from the logistic regression are resented as odds ratios (ORs) with 95% CIs and *p* values. Predicted probabilities from models were plotted to illustrate change in probability. All analyses were performed in R 3.6.1 (R Foundation for Statistical Computing, Vienna, Austria). *p* values <0.05 are considered statistically significant.

## 3. Results

### 3.1. Socio-demographic characteristics

The respondents’ age and gender patterns were similar in the two countries (Table 2). A larger proportion of the Swedish respondents had a university education (36% Denmark, 45% Sweden), but a larger proportion of Danish respondents had a vocational education compared with their Swedish counterparts (34% Denmark, 13% Sweden). More than half of the respondents in both countries were employed (52% Denmark, 60% Sweden) and approximately one-quarter were retired (27% Denmark, 26% Sweden).

**Table 2:** Socio-demographic characteristics of the respondents.

Variables	Country	
	Denmark, *n* (%)	Sweden, *n* (%)
Age	2,619	2,633
< 25 years	411 (15.7)	285 (10.8)
25–65 years	1,609 (61.4)	1714 (65.1)
> 65 years	599 (22.9)	634 (24.1)
Gender	2,619	2,633
Female	1,394 (53.2)	1,448 (55)
Male	1,225 (46.8)	1,185 (45)
Education	2,601	2,628
High school or lower education	784 (30.1)	1,102 (41.9)
Vocational education after high school	873 (33.6)	337 (12.8)
University education	944 (36.3)	1,189 (45.2)
Occupation	2,616	2,626
Employed	1,354 (51.8)	1,577 (60.1)
Student or internship	368 (14.1)	250 (9.5)
Unemployed or long-term sick leave	177 (6.8)	106 (4)
Retired	717 (27.4)	693 (26.4)

### 3.2. Correlation of general acceptability and total score of the seven acceptability items

The correlation between the response to the general acceptability item and the total score for the seven acceptability items ranged from 52 to 69% and was slightly higher for Denmark than for Sweden (Table 3).

**Table 3:** Correlation between the response to the general acceptability item and the total score for the seven acceptability items.

Country	Wave	Correlation (CI)
Denmark	1	0.64 (0.62–0.66)
Denmark	2	0.69 (0.67–0.71)
Sweden	1	0.56 (0.53–0.58)
Sweden	2	0.52 (0.49–0.55)

CI, confidence interval.

### 3.3. Acceptability of the COVID-19 restrictions

**Figure 1:**
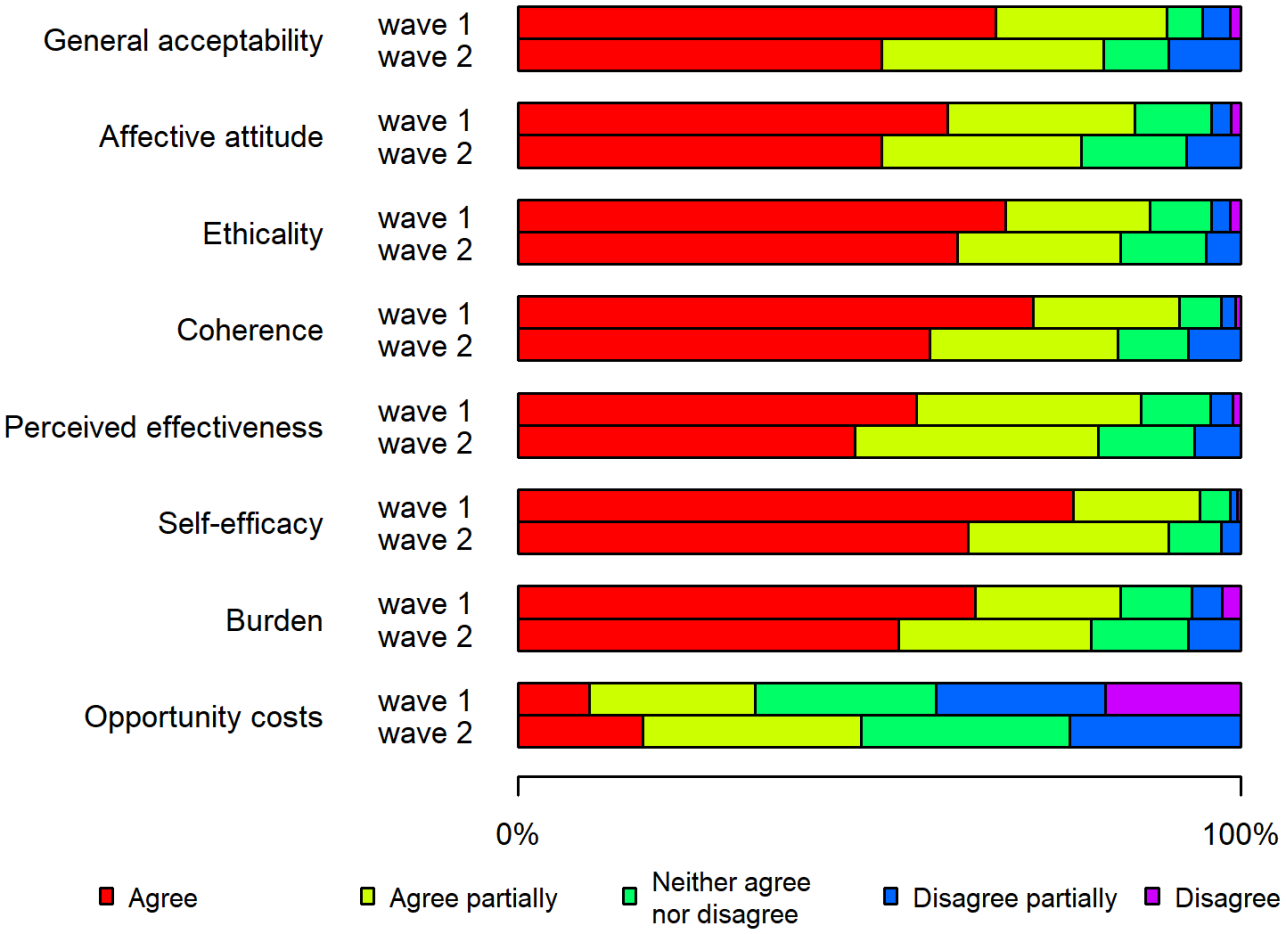
Stacked bar chart showing the acceptability of restrictions in Denmark during the two waves of the pandemic.

**Figure 2:**
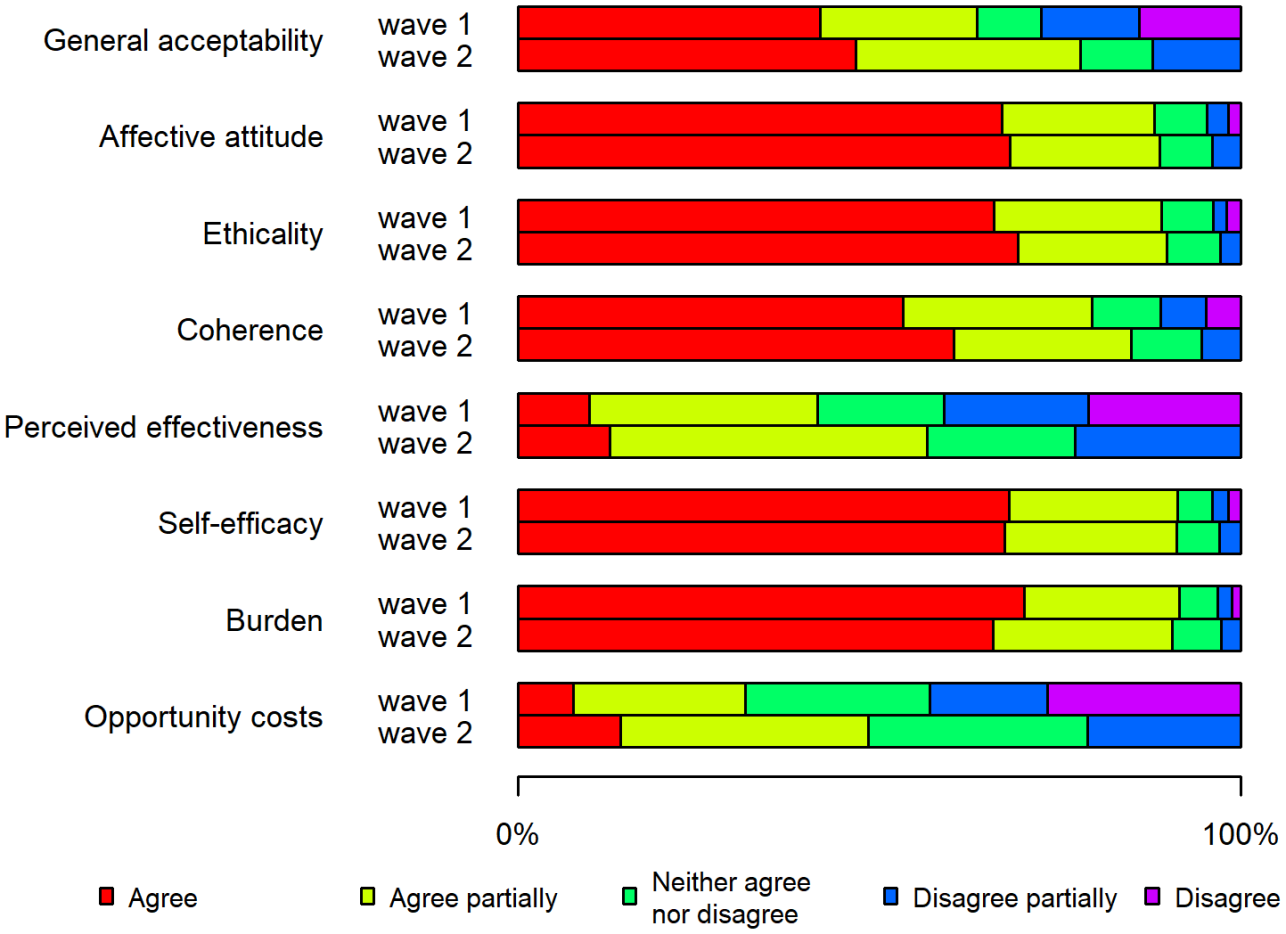
Stacked bar chart showing the acceptability of restrictions in Sweden during the two waves of the pandemic.

The proportion of respondents in Denmark who agreed with the statements (“agree” alternative) that captured various acceptability constructs was generally higher for the first wave (66%) than the second wave (50%) of the pandemic (Figure 1). The opposite pattern was seen for Sweden, where most acceptability statements had higher proportions of agreement (“agree” alternative) for the second wave (47%) compared to the second wave (42%), although the differences were generally small (Figure 2). In both countries, opportunity costs increased in the second wave (Figures 1, 2).

In the first wave, the Danish respondents to a larger degree than the Swedish agreed (“agree” alternative) with most of the statements that captured various acceptability components (Figures 1, 2). Two exceptions to this pattern were the reversed burden construct (63% Denmark, 70% Sweden) and the affective attitude construct (59% Denmark, 67% Sweden).

In the first wave, a few acceptability constructs stood out with regard to differences between Denmark and Sweden (Figures 1, 2): perceived effectiveness (55% Denmark, 10% Sweden) and coherence (71% Denmark, 53% Sweden). In addition, a notably higher proportion of Danish respondents expressed agreement with the general acceptability statement (66% Denmark, 42% Sweden).

In Denmark, the highest proportions of agreement in the first wave were for the constructs of self-efficacy (77%), coherence (71%) and ethicality (67%) (Figure 1). The response pattern was partially different in Sweden in the first wave, where the largest share of agree responses was for the reversed construct burden (70%), followed by self-efficacy (68%) and affective attitude (67%) (Figure 2).

With regard to the second wave, Swedish respondents to a greater extent than the Danish stated that they agreed (“agree” alternative) with most of the statements (Figures 1, 2). There was a particularly large difference seen with regard to affective attitude (48% Denmark, 67% Sweden).

The highest degree of acceptance in the second wave in Denmark was noted for the constructs of self-efficacy (62%), ethicality (59%) and coherence (55%), that is, the same three constructs that had the highest proportions of acceptance in relation to the first wave (Figure 1). The Swedish respondents expressed the highest degree of acceptance for the constructs of ethicality (68%), affective attitude (67%) and self-efficacy (66%) (Figure 2).

### 3.4. Characteristics of low accepters concerning the COVID-19 restrictions

Age was a factor with regard to low accepters, that is, the 25% with the lowest total score on the seven acceptability statements (Table 4). In both waves, younger respondents (<25 and 25–65 years) were more likely than the older respondents (>65 years) to be low accepters. This was particularly notable in the second wave, when Danish respondents aged <25 years were almost eight times more likely than the older respondents (>65 years) to be low accepters.

**Table 4:** Logistic regression of being a low-accepter.

Variable	Comparison	Denmark wave 1		Denmark wave 2		Sweden wave 1		Sweden wave 2	
		OR (CI)	*p* value	OR (CI)	*p* value	OR (CI)	*p* value	OR (CI)	*p* value
Age	<25 years vs. >65 years	3.90 (2.93–5.21)	<0.001	7.67 (5.6–10.7)	<0.001	2.83 (2.11–3.82)	<0.001	5.48 (3.94–7.68)	<0.001
	25–65 years vs. >65 years	1.95 (1.55–2.49)	<0.001	2.94 (2.2–3.93)	<0.001	1.81 (1.48–2.24)	<0.001	2.77 (2.15–3.61)	<0.001
Gender	Male vs. female	1.59 (1.34–1.89)	<0.001	1.30 (1.09–1.55)	0.003	1.33 (1.13–1.56)	<0.001	1.30 (1.10–1.55)	0.003
Education	High school or lower education vs. university	1.50 (1.23–1.85)	<0.001	1.40 (1.14–1.73)	0.001	1.47 (1.23–1.75)	<0.001	1.45 (1.20–1.76)	<0.001
	Vocation vs. university	0.87 (0.71–1.08)	0.203	0.71 (0.57–0.88)	0.002	1.56 (1.21–2.00)	<0.001	1.33 (1.01–1.75)	0.040
Occupation	Employed vs. retired	1.78 (1.43–2.22)	<0.001	2.50 (1.96–3.21)	<0.001	1.86 (1.52–2.28)	<0.001	2.83 (2.21–3.64)	<0.001
	Student or internship vs. retired	3.18 (2.41–4.20)	<0.001	5.43 (4.04–7.34)	<0.001	2.51 (1.85–3.41)	<0.001	4.61 (3.29–6.47)	<0.001
	Unemployed vs. retired	1.86 (1.28–2.68)	0.001	2.60 (1.75–3.83)	<0.001	2.13 (1.39–3.26)	<0.001	2.62 (1.60–4.21)	<0.001

Male respondents were more likely than female respondents to be low accepters concerning the restrictions in both Denmark and Sweden and during both waves. Education also differed with regard to acceptability of the restrictions. Respondents with a high school or lower education were more likely than those with university studies to be low accepters in both countries and in both waves. Respondents with vocational education after high school were less likely than university-educated respondents to be low accepters in Denmark (although not statistically significant in the first wave), but this was not the case in Sweden.

Compared with retired respondents, students and those on internships in both countries and during both waves were most likely to be low accepters concerning the restrictions. This pattern was even more pronounced in the second wave in both countries, when this group was approximately five times more likely than retired respondents to be low accepters. Employed respondents and those who were unemployed or on long-term sick leave were also more likely than retired respondents to be low accepters in both countries and in both waves.

## 4. Discussion

The aim of this study was to investigate the acceptability of the COVID-19 restrictions in Denmark and Sweden concerning the first two waves of the pandemic. Restrictions that are deemed acceptable are more likely to be complied with than those considered unacceptable (8). The respondents in Denmark expressed a higher degree of acceptability with the restrictions in the first wave; the opposite was seen in Sweden. In the first wave, the use of laws and executive orders allowed Denmark to adopt many mandatory restrictions, whereas Sweden relied predominantly on voluntary recommendations until the introduction of a new law in response to the second wave that enabled more rigorous mandatory restrictions. The responses for two countries were more similar during the second wave (4).

The differences between the two countries and between the two waves suggest that there was considerable acceptance for the more strict restrictions because Swedish respondents expressed higher acceptance for the second-wave restrictions. This was also mirrored in the perceived effectiveness of the restrictions, with far higher proportions of respondents in Denmark than in Sweden agreeing. Sweden’s “softer” response in the first wave received a great deal of criticism; it was labelled a herd immunity approach although this was never an officially declared “strategy” (6). Sweden had far higher per-capita incidence, hospitalization and mortality rates due to COVID-19 than Denmark and many other countries, which was a key factor in the rapidly developed law and new approach (5).

The survey findings also showed that respondents in Denmark expressed lower acceptance of the restrictions in the second wave despite the fact that they were broadly similar to those used in the first wave. The difficulty of maintaining restrictions over longer periods was something that experts at the Public Health Agency in Sweden addressed. The risk of weariness with long-term restrictions was a key argument for the agency’s emphasis on less severe restrictions in the first wave (6). The Danish public seemed to experience an increased weariness to comply with the restrictions over time. This interpretation is strengthened by the fact that the opportunity cost, i.e., one of the seven acceptability constructs, was higher in the second wave than the first wave in both countries. Thus, the perceived benefits, values or profits the public had to give up for the restrictions increased over time, implying a restriction (or pandemic) fatigue effect. Restriction fatigue has been conceptualized as a state of weariness, exhaustion and reduced motivation to perform various activities, e.g., complying with restrictions due to the pandemic (16). The World Health Organization describes this as a latent phenomenon that is not directly observable, but is expressed on a behavioural level “through an increasing number of people not sufficiently following recommendations and restrictions, [and] decreasing their effort to keep themselves informed about the pandemic” (17, p. 7). A gradual decrease in compliance with restrictions over the course of the pandemic was seen in several countries (18–20).

There were considerable age, gender, education and occupation differences between the low accepters and the other respondents with regard to acceptability of the COVID-19 restrictions. There were similar patterns in the two countries for socio-demographic characteristics of the low accepters, i.e., they were younger, more often male and more likely to have lower education levels. The notable variations imply that crisis communication about the pandemic might benefit from being tailored to different segments of the population to achieve increased acceptability in some population sub-groups. Research on crisis communication, i.e., the collection, processing and dissemination of information to address a crisis situation, emphasizes that communication should be adapted to the target groups (21–23). However, the governments and public authorities in Denmark and Sweden did not use any specific tailored communications to reach different population sub-groups based on shared socio-demographics or other characteristics (24, 25). The authorities in both countries received considerable critique for insufficient adaptation of communications to many immigrant groups beyond translation of messages into multiple languages (26, 27). The challenges of reaching some immigrant groups in both countries became manifest when the COVID-19 vaccination programmes rolled out, starting in 2021; vaccination rates among some sub-groups of the immigrant populations in both Denmark and Sweden were considerably lower (28).

Age seemed to be a particularly important socio-demographic characteristic that distinguished between low accepters and all others. This finding might be explained with reference to the use of different media. Younger people were less likely than older age groups to rely on traditional news broadcasts and public authorities’ information channels where pandemic-relevant information was predominantly communicated (29). A study in Norway found that young persons aged 13–20 years retrieved most information from the internet and online news, and they perceived this information as sufficient and covering their information needs (30). However, the younger age group’s lower acceptance of the restrictions could also reflect a lower perceived or real risk of getting COVID-19 and becoming seriously ill when infected (31). In general, younger age groups tend to have a lower perception of risk than other age categories (32), which may be explained by the lower morbidity and mortality seen in this age group (33). An Italian study noted that young people aged 13–20 years had a low risk perception of COVID-19 and that they underestimated the probability of getting the disease (34). Lower risk perception likely decreases individuals’ acceptance of and compliance with restrictions.

A relatively low correlation was seen between the response to the general acceptability item and the total score for the seven acceptability items. This finding suggests that the general item does not capture the comprehensiveness of the multifaceted acceptability concept. The various items range from assessments of negative consequences of the restrictions (e.g., burden and opportunity costs) to more positive aspects of the restrictions (self-efficacy) and from more personal, subjective appraisals (e.g., affective attitude and ethicality) to more objective opinions of the restrictions (e.g., coherence and perceived effectiveness).

This study has some limitations that must be considered when interpreting the results. The questionnaire has been developed in a thorough process using think-aloud methods to achieve comprehensibility, relevance and answerability of the items (14). However, further testing, including of psychometric properties, is required to establish validity and reliability of the instrument. The study was a cross-sectional survey, based on self-reports. Surveys are usually associated with some response bias, i.e., various conditions that can influence responses and make survey data less useful. The panels used by Enkätfabriken (the survey company in charge of the data collection) are nationally representative on socio-demographic variables, but we do not know their motivation or interest in responding to the survey about COVID-19. It is possible that those who did respond are the ones most interested in the pandemic and responses taken in either country to reduce the spread, and thus may not be fully representative of the general population in each country. However, it is difficult to assess how this might have affected the results, i.e., whether they were more or less accepting of the restrictions than the broader populations. Additionally, even though the sample is nationally representative, minority groups such as gender and racial minorities might not be very well represented. These limitations notwithstanding, the study also has considerable strengths, including the relatively large sample size for the survey and the comprehensive think-aloud study to ascertain the robustness of the questionnaire. Furthermore, the questionnaire was theoretically grounded in concepts of acceptability that have been applied in earlier studies.

In conclusion, this survey found that respondents in Sweden were more accepting of the restrictions in the second wave, when the country introduced many more mandatory restrictions. In contrast, respondents in Denmark were more accepting of the COVID-19 restrictions in the first wave of the pandemic than in the second wave, implying an increased weariness to comply with the restrictions over time. The opportunity cost was higher in the second wave than the first wave in both countries. There were considerable socio-demographic differences between those who expressed low acceptability of the restrictions and the others in both countries, suggesting the relevance of tailoring communication about the pandemic to different segments of the population.

## Data availability statement

The raw data supporting the conclusions of this article will be made available by the authors, without undue reservation.

## Ethics statement

The project adheres to the directives of the Helsinki Declaration. The participants provided their written informed consent to participate in this study. The studies involving human participants were reviewed and were approved by the Danish Data Protection Agency [P-2021-13] and the Danish Committee System on Health Research Ethics [Journal-no.: 20052405].

## Author contributions

PN, IS, NS, JK, OA, TT-T, TK, MS, and KV: conceptualization. PN, IS, NS, and JK: methodology. PN, TK, and MS: formal analysis. PN and JK: investigation, resources, writing—original draft preparation, writing—review and editing, and supervision and project administration. OA, JK, and PN: funding acquisition. All authors contributed to the article and approved the submitted version.

## Funding

This work was supported by Innovation Fund Denmark (0211-00026B).

## Conflict of interest

The authors declare that the research was conducted in the absence of any commercial or financial relationships that could be construed as a potential conflict of interest.

## Publisher’s note

All claims expressed in this article are solely those of the authors and do not necessarily represent those of their affiliated organizations, or those of the publisher, the editors and the reviewers. Any product that may be evaluated in this article, or claim that may be made by its manufacturer, is not guaranteed or endorsed by the publisher.

## References

[1] Esping-Andersen G. The three worlds of welfare capitalism. Cambridge, UK: Polity Press (1990).

[2] Mishra S Scott JA Laydon DJ Flaxman S Gandy A Mellan TA. Comparing the responses of the UK, Sweden and Denmark to COVID-19 using counterfactual modelling. Sci Rep. (2021) 11:16342. doi: 10.1038/s41598-021-95699-9 34381102 PMC8358009

[3] Marin C. Europe versus coronavirus—Putting the Danish model to the test. Paris: Institut Montaigne (2020).

[4] Seing I Stefansdottir NT Kirk JW Andersen O Tjörnhöj-Thomsen T Kallemose T. Social distancing policies in the coronavirus battle: a comparison of Denmark and Sweden. Int J Environ Res Public Health. (2021) 18:10990. doi: 10.3390/ijerph182010990, PMID: 34682734 PMC8536108

[5] Coronavirus Resource Center. John Hopkins University of Medicine (2020/2021). Available at: https://coronavirus.jhu.edu/map.html (Accessed October 17, 2021).

[6] Anderberg J. Flocken—Berättelsen om hur Sverige valde väg under Pandemin [Flocken — The story of how Sweden chose the path during the pandemic]. Stockholm: Albert Bonniers Förlag (2021).

[7] Anand U Cabreros C Mal J Ballesteros F Jr Sillanpää M Tripathi V. Novel coronavirus disease 2019 (COVID-19) pandemic: from transmission to control with an interdisciplinary vision. Environ Res. (2021) 197:111126. doi: 10.1016/j.envres.2021.111126, PMID: 33831411 PMC8020611

[8] Sekhon M Cartwright M Francis JJ. Acceptability of healthcare interventions: an overview of reviews and development of a theoretical framework. BMC Health Serv Res. (2017) 17:88. doi: 10.1186/s12913-017-2031-8, PMID: 28126032 PMC5267473

[9] Sekhon M Cartwright M Francis JJ. Acceptability of health care interventions: a theoretical framework and proposed research agenda. Br J Health Psychol. (2018) 23:519–31. doi: 10.1111/bjhp.12295, PMID: 29453791

[10] Jörgensen F Bor A Petersen MB. Compliance without fear: individual-level protective behaviour during the first wave of the COVID-19 pandemic. Br J Health Psychol. (2021) 26:679–96. doi: 10.1111/bjhp.12519, PMID: 33763971 PMC8250211

[11] Lunn PD Timmons S Belton CA Barjakova M Julienne H Lavin C. Motivating social distancing during the COVID-19 pandemic: an online experiment. Soc Sci Med. (2020) 265:113478. doi: 10.1016/j.socscimed.2020.113478, PMID: 33162198

[12] Enkätfabriken. Undersökningar som leder till samhällsutveckling! [Surveys that Lead to Societal Development] (2022). Available at: https://www.enkatfabriken.se/ (Accessed May 31, 2022).

[13] von Elm E Altman DG Egger M Pocock SJ Gøtzsche PC Vandenbroucke JP. The strengthening the reporting of observational studies in epidemiology (STROBE) statement: guidelines for reporting observational studies. Lancet (London, England). (2007) 370:1453–7. doi: 10.1016/S0140-6736(07)61602-X 18064739

[14] Gardner B Tang V. Reflecting on non-reflective action: an exploratory think-aloud study of self-report habit measures. Br J Health Psychol. (2013) 19:258–73. doi: 10.1111/bjhp.12060 23869847 PMC4296343

[15] Sekhon M Cartwright M Francis JJ. Development of a theory-informed questionnaire to assess the acceptability of healthcare interventions. BMC Health Serv Res. (2022) 22:279. doi: 10.1186/s12913-022-07577-3 35232455 PMC8887649

[16] Lilleholt L Zettler I Betsch C Böhm R. Pandemic fatigue: measurement, correlates and consequences. *PsyArXiv* [Epub ahead of preprint] (2021). 1–36. doi: 10.31234/osf.io/2xvbr

[17] World Health Organization. Pandemic fatigue. Copenhagen: WHO (2020). Available at: https://apps.who.int/iris/bitstream/handle/10665/335820/WHO-EURO-2020-1160-40906-55390-eng.pdf (Accessed May 31, 2022).

[18] Petherick A Goldszmidt R Andrade EB Furst R Hale T Pott A. A worldwide assessment of changes in adherence to COVID-19 protective behaviours and hypothesized pandemic fatigue. Nat Hum Behav. (2021) 5:1145–60. doi: 10.1038/s41562-021-01181-x, PMID: 34345009

[19] Wright L Steptoe A Fancourt D. Trajectories of compliance with COVID-19 related guidelines: longitudinal analyses of 50, 000 UK adults. *medRxiv* [Epub ahead of preprint] (2021) doi: 10.1101/2021.04.13.21255336 PMC9278256 35759288

[20] MacIntyre CR Nguyen PY Chughtai AA Trent M Gerber B Steinhofel K. Mask use, risk-mitigation behaviours and pandemic fatigue during the COVID-19 pandemic in five cities in Australia, the UK and USA: a cross-sectional survey. Int J Infect Dis. (2021) 106:199–207. doi: 10.1016/j.ijid.2021.03.056, PMID: 33771668 PMC7985682

[21] Renn O. Risk governance: Coping with uncertainty in a complex world. London: Earthscan (2008).

[22] Renn O. The call for sustainable and resilient policies in the Covid-19 crisis. How can they be interpreted and implemented? Sustainability. (2020) 12:6466. doi: 10.3390/su12166466

[23] Bouder F. Principles and challenges of risk communication/crisis communication, specifically addressing issues relating to pandemics. Underlagsrapport till SOU 2022: 10 Sverige under pandemin. Stockholm: SOU (2022).

[24] Diaz E Norredam M Aradhya S Benfield T Krasnik A Madar A. Situational brief: migration and Covid-19 in Scandinavian countries, December 18th 2020 Lancet Migration (2020). Available at: https://www.migrationandhealth.org/migration-covid 19-briefs (Accessed June 12, 2022).

[25] Nielsen JH Lindvall J. Trust in government in Sweden and Denmark during the COVID-19 epidemic. West Eur Polit. (2021) 44:1180–204. doi: 10.1080/01402382.2021.1909964

[26] Stjernswärd S Ivert A-K Glasdam S. Perceptions and effects of COVID-19 related information in Denmark and Sweden – a web-based survey about COVID-19 and social media. Z Gesundh Wiss. (2021) 26:1–15. doi: 10.1007/s10389-021-01539-5 PMC8071611 33936931

[27] Sigurjonsdottir HR Sigvardsson D Costa SO. Who is left behind? The impact of place on the possibility to follow Covid-19 restrictions. Nordic Council of Ministers: Copenhagen (2021).

[28] European Centre for Disease Prevention and Control. Fremme af tilslutning til og udbredelse af covid-19-vaccination i EU/EØS 15 oktober 2021 [promoting the connection to and spread of covid-19 vaccination in the EU/EEA 15 October 2021]. Stockholm, ECDC: (2021).

[29] Dyregrov A Fjærestad A Gjestad R Thimm J. Young people's risk perception and experience in connection with COVID-19. J Loss Trauma. (2021) 26:597–610. doi: 10.1080/15325024.2020.1853974, PMID: 34065093

[30] Kyrrestad HKS Kaiser S Martinussen M. Ungdoms opplevelse av informasjon som gis om koronaviruset (Covid-19): En foreløpig rapport [Adolescents' experience of information provided about the coronavirus (Covid-19): A preliminary report]. RKBU Nord: UiT Norges arktiske universitet (2020). 26 p.

[31] Savadori L Lauriola M. Risk perception and protective behaviors during the rise of the COVID-19 outbreak in Italy. Front Psychol. (2021) 11:577331. doi: 10.3389/fpsyg.2020.577331, PMID: 33519593 PMC7838090

[32] Reniers RL Murphy L Lin A Bartolome SP Wood SJ. Risk perception and risk-taking behaviour during adolescence: the influence of personality and gender. PLoS One. (2016) 11:e0153842. doi: 10.1371/journal.pone.0153842, PMID: 27100081 PMC4839773

[33] Yang XY Gong RN Sassine S Morsa M Tchogna AS Drouin O. Risk perception of COVID-19 infection and adherence to preventive measures among adolescents and young adults. Children. (2020) 7:311. doi: 10.3390/children7120311, PMID: 33371272 PMC7766485

[34] Commodari E La Rosa VL. Adolescents in quarantine during COVID-19 pandemic in Italy: perceived health risk, beliefs, psychological experiences and expectations for the future. Front Psychol. (2020) 11:559951. doi: 10.3389/fpsyg.2020.559951, PMID: 33071884 PMC7538632

